# Pyoderma Grangrenosum

**DOI:** 10.5811/cpcem.1675

**Published:** 2025-02-26

**Authors:** Reza Aghaei, Edmund Hsu, Christopher McCoy

**Affiliations:** * Kaiser Permanente, Central Valley, Department of Emergency Medicine, Modesto, California; † University of California, Irvine, Department of Emergency Medicine, Orange, California

**Keywords:** Pyoderma gangrenosum, ulcerative colitis

## Abstract

**Case Presentation:**

We describe a middle-aged female with past medical history of ulcerative colitis presenting to the emergency department with bilateral painful ulcers rapidly growing on her lower legs in the prior four weeks. She was consulted by a dermatologist and after a thorough clinical and pathology assessment (as a diagnosis of exclusion), treatment for pyoderma gangrenosum was started.

**Discussion:**

Pyoderma gangrenosum is a painful, chronic, ulcerative disorder often occurring in association with systemic disease. We review the clinical presentation of pyoderma gangrenosum and its complications. We describe the characteristics of ulcers with pictures from the patient. Our case illustrates the findings of pyoderma gangrenosum both clinically and pathologically.

## CASE PRESENTATION

**Image 1:**
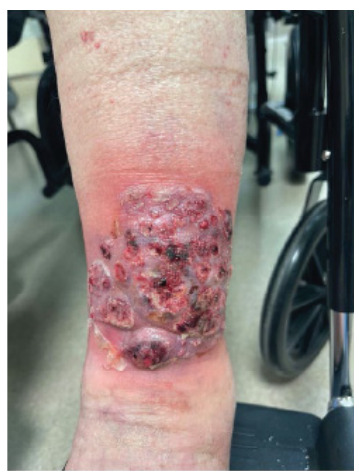
The patients left lower leg ulcer characteristic of pyoderma gangrenosum.

**Image 2:**
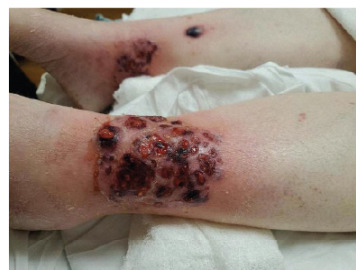
This image shows both of the patients lower leg ulcers demonstrating different stages of pyoderma gangrenosum.

**Image 3:**
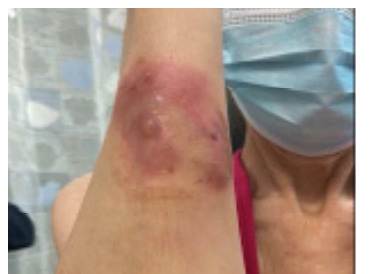
The patients’ right forearm pustular lesion, demonstrating another stage of pyoderma gangrenosum.

A 51-year-old female presented with bilateral painful shin ulcers. She has a medical history of ulcerative colitis and multiple sclerosis, for which she was being treated with glatiramer acetate and mesalamine, respectively. The patient denied experiencing fever, chills, or night sweats. She also had a brown nodular painful ulcer on her right forearm, which presented four weeks prior. Her exam was significant for bilateral lower leg palm-sized cribriform ulcer, large violaceous bullous plaque on her right lower leg, and red-brown small nodular plaque on the right forearm (Images 1–3). The patient’s clinical condition was reviewed by a dermatologist, and after a thorough clinical and pathological assessment, treatment for pyoderma gangrenosum was initiated.

## DISCUSSION

Pyoderma gangrenosum (PG) is a reactive, non-infectious, inflammatory dermatosis and is classified as one of the neutrophilic dermatoses, along with Sweets syndrome and Behcets disease. The incidence of PG is approximately 0.63 per 100,000 people, with the median age of onset being 59 years.^1^

Although the lower legs are the most frequently affected, PG can manifest on any part of the body. The condition is often precipitated by minor trauma, a phenomenon known as “pathergy.”^2^

A thorough history is key with specific inquiry regarding possible pathergic response to minor or major trauma, as well as a history of pain, rapid progression, symptoms suggestive of infection or systemic disease and a detailed drug history. 3 Indeed, PG lesions are all too often misdiagnosed as simple non-healing ulcers and patients undergo debridement, which can result in catastrophic deterioration of the condition through this pathergic response. The condition predominantly affects adults, but childhood cases are rarely reported.^4^ More recently, Mavrakis et al have proposed new criteria based on a consensus of international experts, requiring one major and four minor criteria.^5^

**CPC-EM Capsule**

What do we already know about this clinical entity?

*Pyoderma gangrenosum is a neutrophilic dermatosis, which presents most commonly in patients with inflammatory bowel disease*.

What is the major impact of the image(s)?

*We present the classic presentation of pyoderma gangrenosum regarding images, also in a clinical course, which can assist physicians in making a diagnosis*.

How might this improve emergency medicine practice?

*Recognizing pyoderma gangrenosum classic features and presentation can help physicians increase their index of suspicion and diagnostic accuracy leading to improved outcomes*.
